# Presentation of Dyke-Davidoff-Masson Syndrome in a 32-Year-Old Female: Report of a Rare Case With a Literature Review

**DOI:** 10.7759/cureus.41101

**Published:** 2023-06-28

**Authors:** Muhammad Zubair Khan, Shruti Sagar Mahapatra, Tirath Patel, Waleed Razzaq, Uzzam Ahmed Khawaja

**Affiliations:** 1 Department of Internal Medicine, Ayub Teaching Hospital, Abbottabad, PAK; 2 Department of Internal Medicine, SCB Medical College and Hospital, Cuttack, IND; 3 Department of Medicine, American University of Antigua, St. John's, ATG; 4 Department of Internal Medicine, Services Hospital, Lahore, PAK; 5 Department of Pediatrics and Child Health, Aga Khan University Hospital, Karachi, PAK

**Keywords:** symptomatic management, adult onset ddms, mri, ct scan, calvarial hypertrophy, ventricular dilatation, asymmetric cerebral encephalomalacia, dyke-davidoff-masson syndrome

## Abstract

Dyke-Davidoff-Masson syndrome (DDMS) is a rare congenital or acquired neurological disorder that most commonly affects the pediatric population but is also rarely reported in adults. DDMS results from brain injury in the intrauterine or early years of life. It is characterized by prominent cortical sulci, hyperpneumatization of the frontal sinus, unilateral cerebral hemiatrophy with ventricular dilation, and associated bony thickness of the cranial vault. Seizures and asymmetric hemiparesis are the most consistent findings in DDMS with facial asymmetry and mental retardation widely reported. Herein, we report a case of a 32-year-old female patient with DDMS presenting with a history of seizure and right-sided hemiparesis. Neuroimaging findings showed asymmetric cerebral encephalomalacia and gliosis with ex vacuo ventricular dilatation and calvarial diploic space widening. Our case report is unique in the sense that our patient presented with DDMS in adulthood with no signs of mental retardation or history of seizures during childhood and well-controlled seizures on monotherapy. Given the adult presentation of DDMS is unusual and rarely reported in the medical literature, our case report will help physicians to keep DDMS high on differential diagnoses in such cases. Awareness of the clinical features of DDMS on imaging can facilitate a timely and accurate diagnosis, thereby enabling appropriate and prompt management.

## Introduction

Dyke-Davidoff-Masson syndrome (DDMS) is a rare congenital, neonatal, or early infantile condition which classically involves cerebral atrophy or hypoplasia. It usually occurs due to an insult to the developing brain in intrauterine or early childhood [1]. There are several etiologies of DDMS, including prenatal, perinatal, and postnatal. Prenatal causes include infections, congenital anomalies, and cerebral infarction; perinatal causes include birth trauma, hypoxia, and intracranial bleeding; and postnatal causes include brain trauma, prolonged febrile seizures, infections, and brain tumors [2]. 

DDMS frequently manifests in males and involves one cerebral hemisphere, predominantly the left cerebral hemisphere. The age of presentation depends on the time of the neurological insult, with most cases being diagnosed in either childhood or adolescence due to predominant clinical manifestations. Up until 2018, only 21 cases have been reported where the disease was diagnosed in adulthood [3]. An extensive literature search of databases like PubMed, Google Scholar, etc., traces the existence of DDMS back to as far as the fifteenth-century BC. A recent study conducted in 2018 revealed the discovery of a trepanated skull from the Late Bronze and Early Iron Ages (fifteenth-century BC), found at a burial site in Bardzryal, Armenia. Of note, there was the presence of bone nodules on the endocranial surface of the skull, raising suspicion that the skull belonged to a patient with DDMS [4]. However, the first case of DDMS was first clinically reported in 1933 by Dyke, Davidoff, and Masson when they described cranial asymmetry in a series of nine patients with cerebral hemiatrophy, facial asymmetry, mental retardation, and seizures. Since then, this condition has been termed DDMS [5]. From 1933 onward, only 101 clinical cases of DDMS have been reported in the literature. Of note, 22 of these cases have been reported from India, one from Pakistan, three from China, and 10 from Turkey. 

DDMS has a wide spectrum of clinical presentations. Classically, the disease presents with unilateral upper motor neuron lesion-like findings (contralateral to the involved area of the brain) such as hemiparesis or hemiplegia, a variable degree of facial asymmetry, seizures, mental retardation, and behavioral changes [1]. Sensory loss associated with hemiparesis has also been reported [6]. Patients may also have speech or language disorders. Occasionally, patients may have nonspecific symptoms or no clinical symptoms at all and are diagnosed incidentally due to an unrelated cause [7]. One exceptional case described the accidental findings of DDMS in the donor body of a 75-year-old woman who showed no manifestations of DDMS during her entire lifetime [8].

Diagnosis of DDMS is based on the combination of clinical and imaging findings. The diagnostic workup includes electroencephalography (EEG) (in patients who present with seizures) and radiographic studies using brain CT scans and MRI, which are the gold standard. Early manifestations of the disease are often missed on brain CT, and therefore, an MRI brain needs to be done in all patients with suspected DDMS [9]. Brain MRI shows the involvement of brain parenchyma, most commonly cerebral atrophic changes with loss of volume and gliosis, and prominent sulci with homolateral hypertrophy of the paranasal sinuses and skull. A PET brain scan can show hypometabolism of the involved atrophic hemisphere [10].

No standardized management of DDMS exists, and it is mostly symptomatic. The disease management for adult-onset DDMS is different from childhood DDMS [10]. Interventions can range from medications, physiotherapy, speech therapy, and occupational therapy to a surgical approach. Psychotherapy and other alternative treatments, such as ketogenic diets and neurostimulation, may have a role as well [10]. The seizure-predominant disease is hard to control, and the mainstay of treatment stays anti-seizure medications [11]. Insufficient data exist on the prognosis of the disease. However, the disease has shown a favorable prognosis when hemiparesis occurs after the age of two years and in the absence of recurrent seizures [12].

## Case presentation

We present the case of a 32-year-old female who presented to our medicine outpatient department (OPD) with a history of an episode of generalized tonic-clonic seizure (GTCS) in the past 24 hours. She initially had an episode of an unprovoked seizure one year back, following which she was on Levetiracetam but discontinued the medication a month ago. The patient’s past medical history was significant for a stroke resulting in right-sided hemiparesis at the age of 2.5 years, with residual right-sided weakness. Upon admission to the medicine ward, the patient was provisionally diagnosed with a seizure disorder.

**Figure 1:**
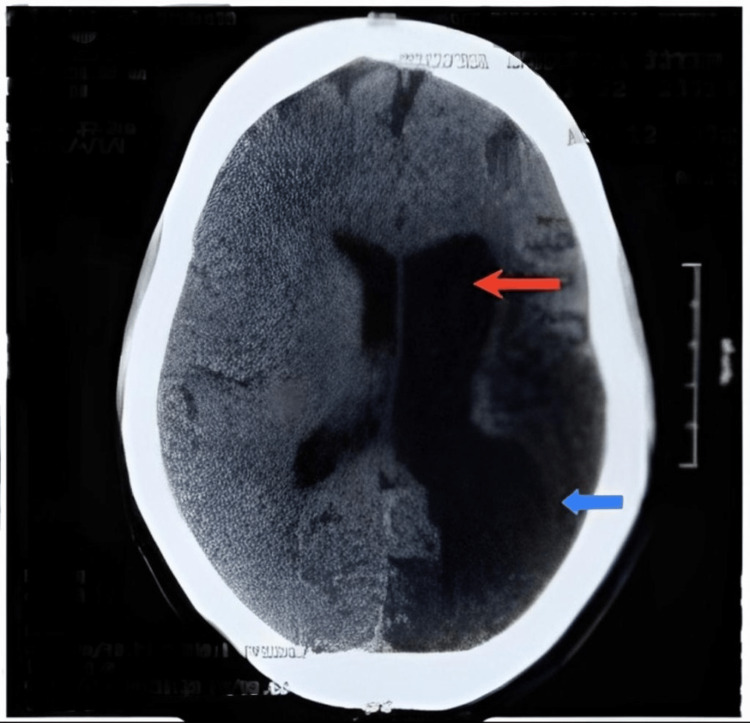
Brain CT scan findings of the patient. The red arrow shows ventricular dilatation of the left lateral ventricle. The blue arrow shows gliotic changes in the temporoparietal and occipital lobes. CT, computed tomography

The patient’s vital signs were within normal limits. She was conscious and oriented. Her higher mental function was normal, and her sensory function appeared to be intact. However, she displayed reduced bulk and power on the right side, as well as hypertonia and a plantar extensor response on the same side. In addition, she exhibited a circumduction gait. Brain CT scan results demonstrated left temporoparietal and occipital gliotic changes, likely related to the patient’s prior stroke, along with ex vacuo dilatation of the ipsilateral ventricle (Figure 1).

 

**Figure 2:**
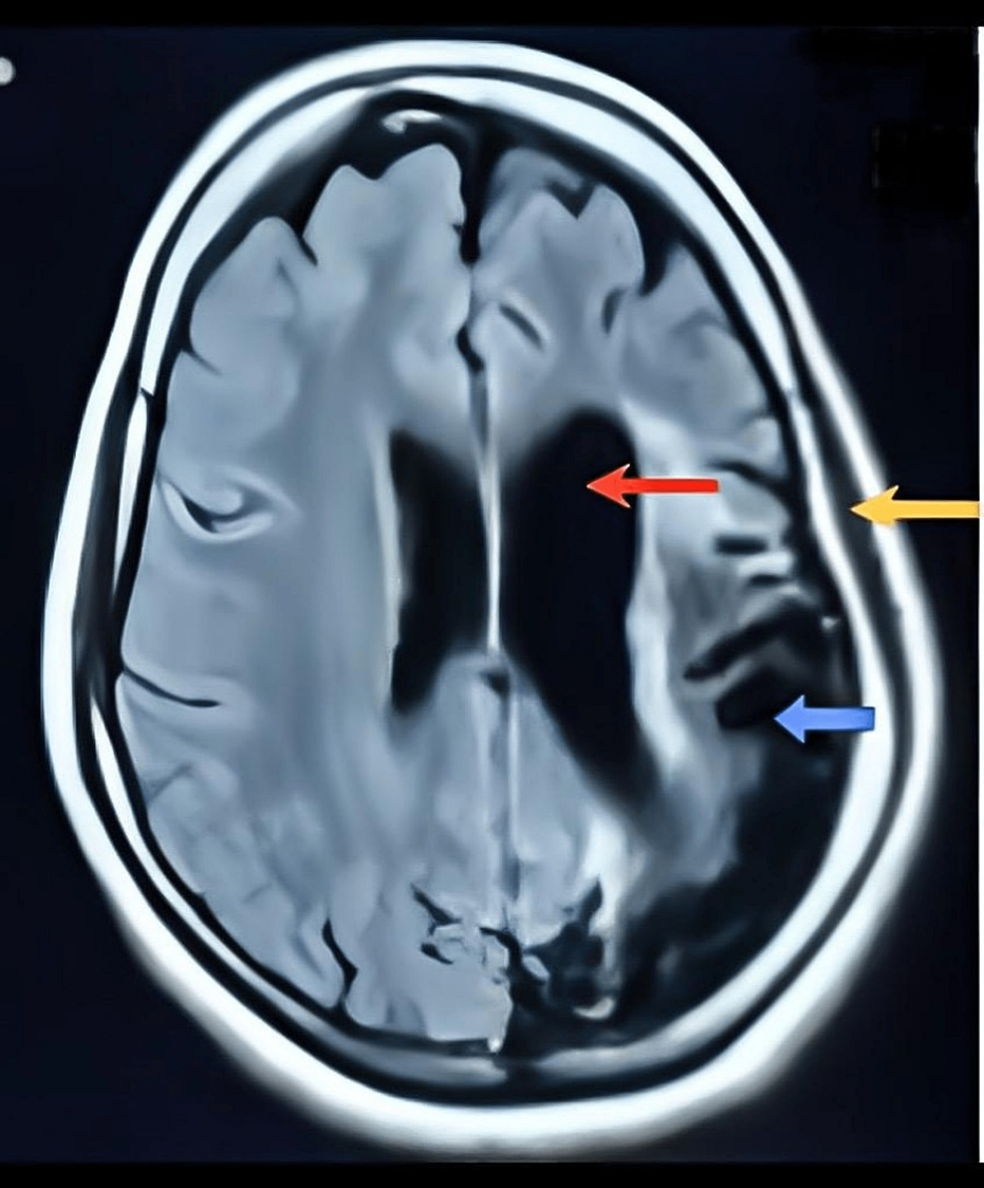
MRI brain scans of the patient. The red arrow shows ventricular dilatation of the left lateral ventricle. The blue arrow shows gliotic changes in the left temporoparietal and occipital lobes. The yellow arrow shows calvarial diploic space widening. MRI, magnetic resonance imaging

Further evaluation with magnetic resonance imaging (MRI) of the brain revealed encephalomalacia and gliosis in the left temporoparietal and occipital lobes with ex vacuo dilatation of the lateral ventricle and calvarial diploic space widening in the left parietal bone and the occipital bone, as shown in Figure 2. Such are the features suggestive of DDMS. The patient’s seizure was managed with a suitable anticonvulsant such as Levetiracetam since the patient had already been in remission with the same drug in the past. For long-term management, physiotherapy and speech therapy were advised.

The patient’s routine blood workup revealed an unremarkable lipid profile along with normal liver and kidney function tests. A sickling test was performed on the blood sample using high-performance liquid chromatography (HPLC), which yielded a negative result. The significant findings of the blood investigation are mentioned in Table 1.

**Table 1:** Remarkable laboratory findings. WBC, white blood cell; RBC, red blood cell

Remarkable investigations	Laboratory findings	Normal values
WBC	15,370/microL	4,000-11,000/microL
Neutrophils	12,190/microL	2,000-8,000/microL
Lymphocytes	2,700/microL	1,500-4,000/microL
Monocytes	370/microL	200-800/microL
Eosinophils	70/microL	40-400/microL
Basophils	40/microL	00-100/microL
Immature granulocytes	70/microL	00-60/microL
RBC	4.88 millions/microL	3.5-5.5 millions/microL
Hemoglobin	12.3 g/dL	11-17 g/dL
Platelets	628,000/microL	150,000-450,000/microL

Soon after discharge the patient returned to a private clinic for a follow-up appointment and exhibited progress. The only reported symptom was a mild headache and the patient was stable overall. It was noted that she had been consistent in taking her prescribed medications as directed.

## Discussion

DDMS was first reported in the literature in 1933 by Dyke, Davidoff, and Masson when they studied a series of nine patients with unique disease features. A literature search showed the predominance of DDMS in childhood [2,9,13]. Only 21 cases have been reported in adulthood, and our case will potentially be the 22nd as we report a 32-year-old woman with clinical features of DDMS. Our patient presented with an episode of GTCS. On examination, she had residual right-sided hemiparesis from a stroke at age 2.5 years involving the left cerebral hemisphere - the same hemisphere involvement seen in around 70% of cases of DDMS [14]. 

In contrast to most cases of DDMS that present with a history of multiple seizure episodes [2,15], our patient had only one seizure episode throughout her lifetime that occurred a year before the current presentation. Most patients who presented with DDMS in adulthood had a seizure onset in early childhood [15,16]. In contrast, our patient had the first seizure episode in adulthood at the age of 31 years. The literature search shows that only two other cases of DDMS in adults had the same presentation with first-time seizure episodes occurring in adulthood [3,17]. Seizures can be focal or generalized. Focal partial seizures with secondary generalization have been reported [3]. Many patients with DDMS reported in adulthood had some degree of mental retardation at presentation, in addition to focal neurologic deficits [16,18,19]. In contrast, our patient had no mental retardation on examination. Some unique cases of DDMS have presented with psychiatric comorbidities like behavioral disturbance and substance use disorder, as described by Bhandarı et al. [20], and schizophrenia, as described by Puri et al. [21]. On the other hand, some cases have been diagnosed incidentally with no clinical manifestations of DDMS [7].

Our patient was diagnosed with DDMS using a brain CT scan, which showed left temporoparietal and occipital gliotic changes, and a brain MRI, which is of great importance in DDMS diagnosis, which showed gliosis in the left temporoparietal and occipital lobes, along with ex vacuo dilation of the left lateral ventricle and widening of the diploic space in the parietal and occipital bones. This was consistent with the radiologic findings found in many other patients with DDMS [22]. A study by Gupta et al. reported unique brain MRI findings of the cerebral peduncle (midbrain) and cerebellar atrophy, in addition to cerebral hemiatrophy with dilated sulci and left calvarial thickening, in a 14-year-old boy with DDMS [23], which were seen in our patient as well. Shen et al. identified three types of MRI patterns that indicate cerebral hemiatrophy [24]. The pattern I on MRI shows widespread atrophy in both the cortical and subcortical areas, while pattern II represents cortical atrophy and porencephalic cysts, and pattern III shows gliosis resulting from previous infarction in the middle cerebral artery region [24]. Our patient’s MRI scan showed pattern III, as described by Shen et al. [24].

The dilemma is that there are no definitive treatment guidelines for seizure management in DDMS patients. Various antiepileptic drugs are used either alone or in combination. Patients have responded differently to different antiepileptics. Bhol et al. described two cases where both DDMS patients with focal seizures responded differently to the same anticonvulsant therapy [25]. Al-Smair et al. reported a 55-year-old DDMS female patient with GTCS who was started on a single antiepileptic drug, valproic acid, although no mention of the treatment efficacy was made in this case report [22]. In comparison, our patient was also started on a single antiepileptic drug, levetiracetam, to control the seizures to which she responded well without requiring any dose adjustments. This contrasts with some cases of DDMS where patients required a combination of two antiepileptics and dose adjustments for seizure control. Al-Attas and Alwazna [10] described a 29-year-old DDMS patient with tonic-clonic seizures, who required an escalating dose of levetiracetam with a standard dose of carbamazepine (600 mg twice daily) to control the seizures. Similarly, a combination of magnesium valproate and carbamazepine was shown to reduce the frequency and duration of seizures in a 24-year-old female patient with DDMS [26]. For patients, especially children, with hemiplegia and recurrent seizures not responding to pharmacotherapy, hemispherectomy is the treatment of choice [2]. Hemispherectomy has been successful in 85% of appropriately selected patients [11]. In general, if the patient's presentation is late, as in our case, and if seizures are well-controlled with pharmacotherapy, it is better to avoid undergoing surgery. Quality of life can further be improved with physiotherapy, speech therapy, and occupational therapy, which are standard management tools in such cases.

Because of its clinical uniqueness and wide spectrum of presentation, the prognosis of DDMS is unclear and depends on how early the disease is diagnosed. Early recognition of the disease with proper intervention has a better prognosis [27]. The disease has poor outcomes in cases of prolonged seizure episodes and hemiparesis. In their case report of a six-year-old boy with DDMS, Sharma et al. showed that the disease had a favorable outcome with the antiepileptic medications [28]. If motor difficulties arise after two years of age and seizures can be controlled, the disease has a favorable prognosis [11]. Based on this information, we can conclude that our patient’s long-term prognosis is favorable. Since the data on the disease management and subsequent prognosis is limited, there is a need to conduct further longitudinal studies to better understand the prognosis of the disease.

## Conclusions

DDMS is a neurologic disorder characterized by a constellation of symptoms of varying severity. Adult presentation of DDMS is a rare finding with no relevant pediatric history. As this is a very rare disorder, it can easily go undiagnosed by the inexperienced eye. Management is variable and depends on the age of the presentation and the severity of the symptoms. This case report of an adult patient with DDMS with a history of seizure and hemiparesis and no other significant findings of DDMS emphasizes the importance for physicians to consider DDMS as a potential diagnosis in such cases, in addition to managing the seizures with monotherapy. Powerful imaging studies like brain CT and MRI are thoroughly advised when attending to patients with clinical features like DDMS to confirm the diagnosis. Larger longitudinal studies are advised to better understand the management options for DDMS and the subsequent prognosis.
